# Identification of clinically relevant patient endotypes in traumatic brain injury using latent class analysis

**DOI:** 10.1038/s41598-024-51474-0

**Published:** 2024-01-14

**Authors:** Hongbo Qiu, Zsolt Zador, Melissa Lannon, Forough Farrokhyar, Taylor Duda, Sunjay Sharma

**Affiliations:** 1 Michael G. DeGroote School of Medicine, McMaster University, Hamilton, ON Canada; 2 Division of Neurosurgery, McMaster University, Hamilton, ON Canada; 3 Department of Health, Evidence and Impact, McMaster University, Hamilton, ON Canada

**Keywords:** Diseases, Health care, Medical research, Neurology, Risk factors

## Abstract

Traumatic brain injury (TBI) is a complex condition where heterogeneity impedes the advancement of care. Understanding the diverse presentations of TBI is crucial for personalized medicine. Our study aimed to identify clinically relevant patient endotypes in TBI using latent class analysis based on comorbidity data. We used the Medical Information Mart for Intensive Care III database, which includes 2,629 adult TBI patients. We identified five stable endotypes characterized by specific comorbidity profiles: Heart Failure and Arrhythmia, Healthy, Renal Failure with Hypertension, Alcohol Abuse, and Hypertension. Each endotype had distinct clinical characteristics and outcomes: The Heart Failure and Arrhythmia endotype had lower survival rates than the Renal Failure with Hypertension despite featuring fewer comorbidities overall. Patients in the Hypertension endotype had higher rates of neurosurgical intervention but shorter stays in contrast to the Alcohol Abuse endotype which had lower rates of neurosurgical intervention but significantly longer hospital stays. Both endotypes had high overall survival rates comparable to the Healthy endotype. Logistic regression models showed that endotypes improved the predictability of survival compared to individual comorbidities alone. This study validates clinical endotypes as an approach to addressing heterogeneity in TBI and demonstrates the potential of this methodology in other complex conditions.

## Introduction

Traumatic brain injury (TBI) is a leading cause of death and disability worldwide^1^ with incidence of 0.2–0.5% per year^2^. It is a complex, heterogeneous condition affecting a broad population, with a wide range of clinical presentations and outcomes. Despite advances toward understanding the pathophysiology of TBI^3,4^, outcome-improving treatments remain rare. A potential explanation for this lack of progress is the heterogeneity of TBI, which may underlie the lack or heterogeneity of benefit observed in multiple large-scale TBI trials (CRASH, DECRA, RESCUE-ICP)^5–7^. One of the important frontiers of TBI research is to deconvolve the condition into a spectrum of disorders that manifest differently between patients^8^. Precision medicine aims to address the shortcomings of our current reductionist approach to the treatment of TBI, instead considering clinical characteristics in individual patients. This strategy expects to identify relatively homogenous patient subgroups that share treatment responses. Precision approaches appear well adapted to increasingly data-rich healthcare systems^9^.

The evolution of high-dimensional longitudinal databases and adaptation of data analytics have improved insight into disease heterogeneity^10,11^. Specifically, the analysis of completed clinical trials or molecular datasets on acute respiratory distress syndrome^12^, asthma^13,14^ and sepsis^15^ have identified homogenous subgroups with distinct clinical features, transforming clinical practice. These analyses promoted the concept of patient “endotypes”, defined as patient subgroups that share clinical and/or molecular characteristics. Endotype research on TBI patient subgroups aims to identify homogeneous populations that share diagnostic criteria, outcomes, and treatment response^16,17^. The identification of such relevant endotypes in TBI may enhance our understanding of disease mechanisms, enable targeted therapeutic approaches, and facilitate stratification for randomized controlled trials.

Comorbidity, the coexistence of two or more chronic conditions, is increasingly common secondary to aging populations^18^ with prevalence between 15 and 54%^19^. Within TBI heterogeneity, patient comorbidity is a commonly reported prognostic factor^20–22^. However, comorbidities do not typically arise in patients randomly. Often, they follow distinct patterns of co-occurrence due to underlying pathophysiological mechanisms. Latent class analysis (LCA) is a well-validated unsupervised machine learning algorithm that can be used as an objective way to discover co-occurrence patterns of comorbidities. LCA assumes unobserved (or latent) clusters underlying the data and uses a probabilistic approach to determine the set of clusters that best explains the observed data^23^. Given comorbidity data inputs, these latent clusters represent patient subgroups with similar comorbidity profiles. LCA has been successfully implemented to describe endotypes in ARDS^12^, sepsis^15^, diabetes^24^, and obesity^25^.

In this study, we hypothesize that TBI patient outcomes vary between comorbidity endotypes. Our primary outcome of interest was survival to discharge, while our secondary outcomes included surgical intervention and duration of hospital stay. We leveraged the prospective Medical Information Mart for Intensive Care III (MIMIC-III) dataset to create a study cohort of adult patients with TBI and used LCA to identify comorbidity endotypes with distinct characteristics. These endotypes may help formalize clinical intuition about prognostication of groups of patients, inform recruitment to clinical trials, and contribute towards the foundation for improved treatment of TBI.

## Methods

### MIMIC III database

Patient information on clinical characteristics and outcomes were extracted from MIMIC-III, a large, single-centre database including 38,597 adult (≥ 16 years old) patients admitted to critical care units at, Beth Israel Deaconess Medical Center, a large tertiary care hospital in Boston, MA^26^. TBI patients were identified from MIMIC-III based on International Classification of Diseases, Ninth Edition (ICD-9) codes. Our study cohort was selected based on established ICD-9 diagnostic codes previously shown to capture the diagnosis of TBI and can be found in Supplementary Table 1^27–29^. We extracted data on patient sex, date of birth, admission date, discharge date, discharge status, ICD-9 diagnosis codes (for patient comorbidities), ICD-9 procedure codes, and initial Glasgow Coma Score (GCS). In patients with re-admission, only data from their first admission was used as this represents the acute TBI treatment phase of interest.

### Data variable construction

The initial total GCS score for each patient was used in defining injury severity as mild (GCS 14–15), moderate (GCS 9–13), or severe (GCS 3–8), as per Brain Trauma Foundation Guidelines^30^. Age was stratified into three bins: young (16–39), middle-aged (40–69), and old (70 +). We used previously published procedure codes that capture intracranial pressure monitor placement, craniotomy/craniectomy, and ventriculostomy, to derive a logistical variable for neurosurgical intervention^28^.

### Definition of comorbidities

We used the well-established Elixhauser comorbidity index^31^ to define chronic conditions by implementing an algorithm provided by the authors of the MIMIC-III database^32^. This algorithm groups ICD-9 codes into 30 chronic conditions to define comorbidity categories. The output is a binary matrix reflecting the presence or absence of a morbidity category by patient ID which was used in subsequent analyses.

### Network discovery

We implemented network discovery to identify co-occurrences in morbidities as described previously^11^. Relative risk (RR) of observing a pair of comorbidities affecting the same patient was calculated using the formula described by Hidalgo et al.^33^. The RR is calculated from the number of patients affected by the two comorbidities, study population, and prevalence of each comorbidity (Supplementary Fig. 1). We then constructed a network of associated comorbidities by only including associations over the significance threshold of p < 0.05 to visualize the relationships between various conditions. Within this network graph, each node represents an Elixhauser comorbidity with node size corresponding to its prevalence and each edge between nodes representing an association between comorbidities with the color corresponding to the RR of the pair and the width to the number of co-occurrences. The colormap for RRs was capped at 5.0 to prevent outliers from skewing visualization.

### Latent class analysis and cluster stability

Latent class analysis (LCA) uses structural equation modeling to identify different subgroups (endotypes) within study populations that share certain characteristics. The R package “poLCA”^34^ was used to identify patient endotypes based on the derived 30 Elixhauser comorbidities. The LCA algorithm fits mixture models to the input data and, through optimization steps, determines the latent classes (endotypes) based on the best-fitting model. For optimization, we used previously published guidance^35,36^, including the elbow method and Akaike information criteria (AIC) to find the optimal number of endotypes. This method involves finding the inflection point of the graph and plotting the number of clusters against an evaluation metric (AIC in this case) which can be seen in Supplementary Fig. 2. We used the R package “inflection”^37^ to objectively determine the inflection point to be at five clusters which represents the optimal number of endotypes.

Due to the stochastic nature of implementing LCA, we took the following additional measures to ensure model validity and cluster stability. We repeated the LCA clustering algorithm on the same data to generate 30 sets of five endotypes. The comorbidity distributions of all 150 total endotypes were then analyzed using latent profile analysis (LPA) which, like LCA, looks for clusters, but in continuous variables rather than categorical. This was done via the R package “mClust”^38^ and compressed the 150 endotypes into a smaller number of recurring endotypes which are visualized in Supplementary Fig. 3. We found that the most well-fit models (models with the lowest AIC) had statistically identical endotype comorbidity profiles. Therefore, we decided to use a representative model within these low-AIC models for our subsequent analysis as this model was inferred to contain endotypes with the greatest stability. Resultant endotypes were characterized clinically through consensus agreement among study authors.

### Quantitative analysis/statistics and visualization

Absolute counts with percentages were reported for categorical data and mean with standard deviation (SD) for continuous data. Statistics were completed using R packages “stats”^39^ and “multcomp”^40^ and Python libraries “statsmodels”^41^ and “SciPy”^42^. The primary outcome was defined as survival to discharge and the secondary outcomes as hospital length of stay (LOS), and neurosurgical intervention.

The Pearson Correlation analysis was conducted to assess the collinearity between age and the number of comorbidities as these were the most likely variables to demonstrate correlation. Only a weak correlation between age and number of comorbidities was found so we used multivariable logistic regression to analyze the relationship between our primary outcome of survival to discharge and number of comorbidities while accounting for key prognostic factors: age, sex, and GCS.

Pairwise chi-squared (survival and intervention rates) and t-tests (LOS) were used for the initial comparisons of clinical outcomes across endotypes and P-values were adjusted using the Holm-Bonferroni method. Multivariable logistic regression and Analysis of Covariance (ANCOVA) was used for more in-depth comparisons of clinical outcomes across endotypes: Multivariable logistic regression analysis was used to compare survival to discharge (yes/no) and neurological intervention (yes/no) between comorbidity endotypes (using the “Healthy” (HE) endotype as the reference category) adjusting for age, sex, and GCS. The odds ratios (OR) with 95% confidence intervals (CI) were reported where appropriate. ANCOVA was used to compare hospital LOS between comorbidity endotypes while adjusting for age, sex, and GCS with a Tukey post-hoc for comparisons between each pair combination of endotypes.

To obtain additional analysis resolution for our primary outcome of survival, we stratified patients within endotypes into groups based on age and GCS bins and conducted paired t-tests between the strata of the endotypes.

Additionally, we created the following multivariable logistic regression models to isolate and assess the performance of endotypes in predicting our primary outcome of survival: (1) model 1 using only the 30 Elixhauser comorbidity categories, (2) model 2 using only the 5 derived comorbidity endotypes and, (3) model 3 using both the 30 Elixhauser comorbidity categories and 5 derived comorbidity endotypes. These models were compared using ANOVA with F scores and P-values reported. Area under the curve (AUC) was also calculated for each model using the R package “pROC”^43^. The above analysis was repeated after adding age, GCS, and sex as covariates to models to assess the effect of Elixhauser comorbidities and endotypes on overall survival prediction performance. Logistic regression models and AUC calculations were also completed for a base model that included only age, GCS, and sex as well as models using only these individual variables. Cases with missing data were excluded from statistical analyses. Figures were generated via the R library “ggplot2” and the Python libraries “matplotlib” and “networkX”.

## Results

### Comorbidity characterization

We included 2,629 patients in our analysis. The mean age was 59.0, 61.9% were male, average GCS was 10.4, the mean number of comorbidities was 2.0, and the rate of survival to discharge was 36.7%. The demographics of our study cohort are summarized in Table 1.

**Table 1:** Study demographics of 2,629 included patients.

Population parameter	Value
Mean age	59 ± 23 years
Male sex	1,627 (61.9%)
Mean (Median) GCS	10.4 (11) ± 4.6
Mean (Median) number of comorbidities	2.1 (2) ± 1.8
Survival to discharge	1665 (63.3%)
Neurosurgical intervention	604 (23.0%)
Mean length of stay	9.3 days ± 10.9 days

Parameters of age, sex, Glasgow coma scale (GCS), number of comorbidities, survival rate, intervention rate, and hospital length of stay for the traumatic brain injury cohort. Continuous variables reported as mean ± SD. Categorical variables reported as count with percentage of population.

**Figure 1:**
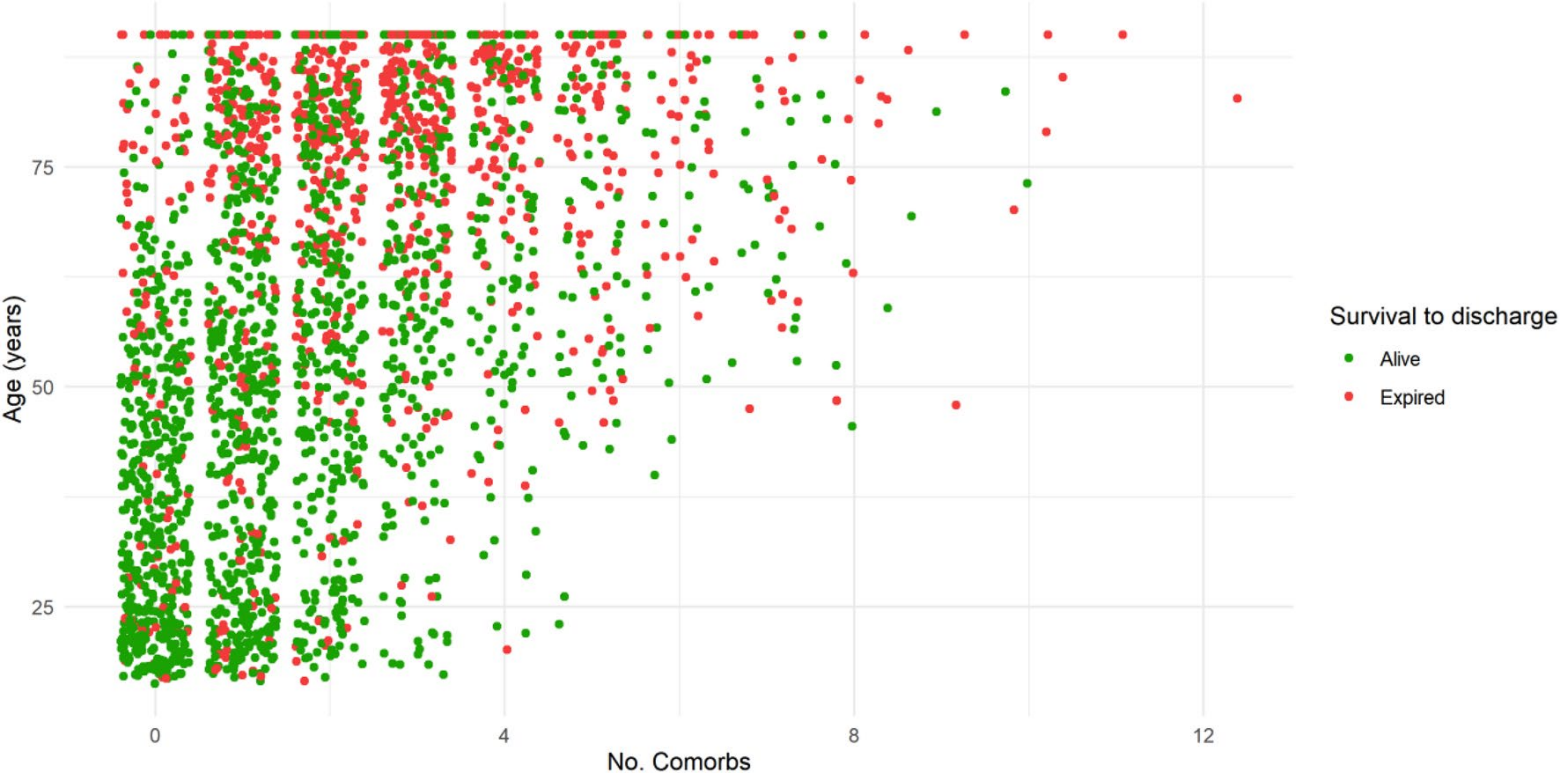
Relationship between age, number of comorbidities, and mortality. Jitter plot of age, number of comorbidities, and survival to discharge across TBI cohort. Both age and number of comorbidities are linearly correlated to survival. Younger patients and patients with lower number of comorbidities tend to have better survival, suggested by the higher proportion of expired patients moving away from the origin of the plot. Age is also correlated with comorbidities as seen by the higher concentration of younger patients at lower numbers of comorbidities and the gradual shift towards older patients with increasing number of comorbidities.

The relationship between age, number of comorbidities, and survival can be visualized in Fig. 1. Age and comorbidity count was only weakly associated (r = 0.20, p < 0.001). We then tested the association of the comorbidity count with the survival to discharge after accounting for age and GCS score in a multivariable logistic regression model and found that the number of comorbidities was negatively associated with survival (OR for survival per one increase in comorbidity count = 0.984, CI: 0.975–0.994).

**Figure 2:**
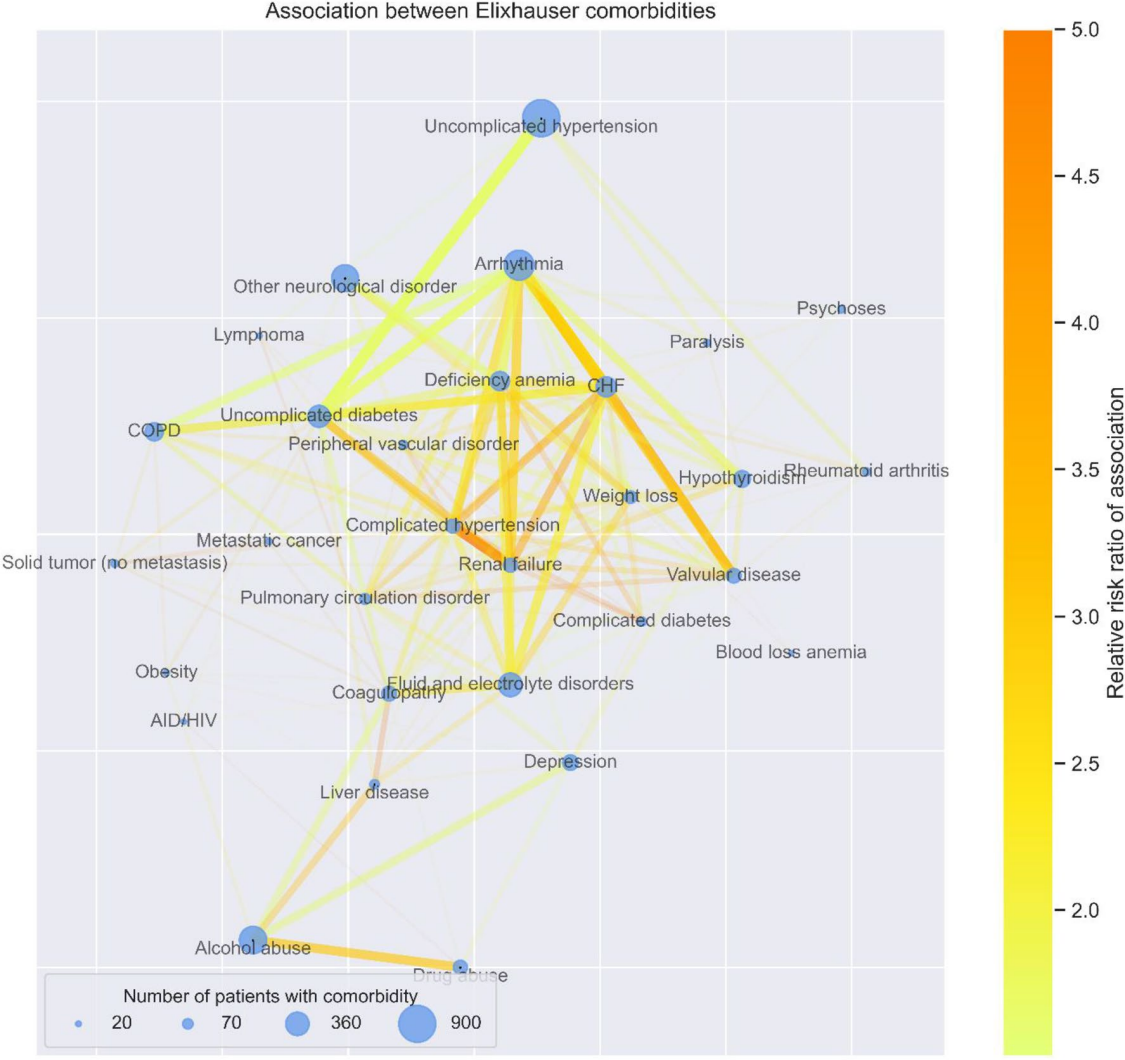
Association between Elixhauser comorbidities. Network graph of Elixhauser comorbidity associations. Each node represents 1 of 30 Elixhauser comorbidity groups. Node size represents prevalence, edge width represents number of patients with specific co-occurrence, edge color represents strength of association in terms of relative risk (RR) calculated as described in the methodology. Many clinically intuitive associations emerge such as congestive heart failure with arrhythmia and valvular diseases, alcohol abuse with drug abuse, and renal failure with hypertension. Only associations with p < 0.05 and RR > 1.5 are shown to reduce cluttering from weaker associations. RR is capped at 5 for visualization purposes (i.e., RRs > 5 were shown only as 5) to prevent very strong associations from skewing the upper range of the color bar.

We next investigated the pattern of comorbidities, specifically their associations in the entire cohort to test our hypothesis that many comorbidities co-occur. These relationships between the Elixhauser comorbidity groups are visualized by the network graph in Fig. 2. This network discovery of the data revealed intuitive subgroups: congestive heart failure (CHF) with arrhythmia (RR = 2.76, CI: 2.73–2.79), drug abuse with alcohol abuse (RR = 3.02, CI: 2.95–3.11), and renal failure with complicated hypertension (RR = 16.27, CI: 15.96–16.58).

### Clinical characterization of comorbidity endotypes

**Figure 3:**
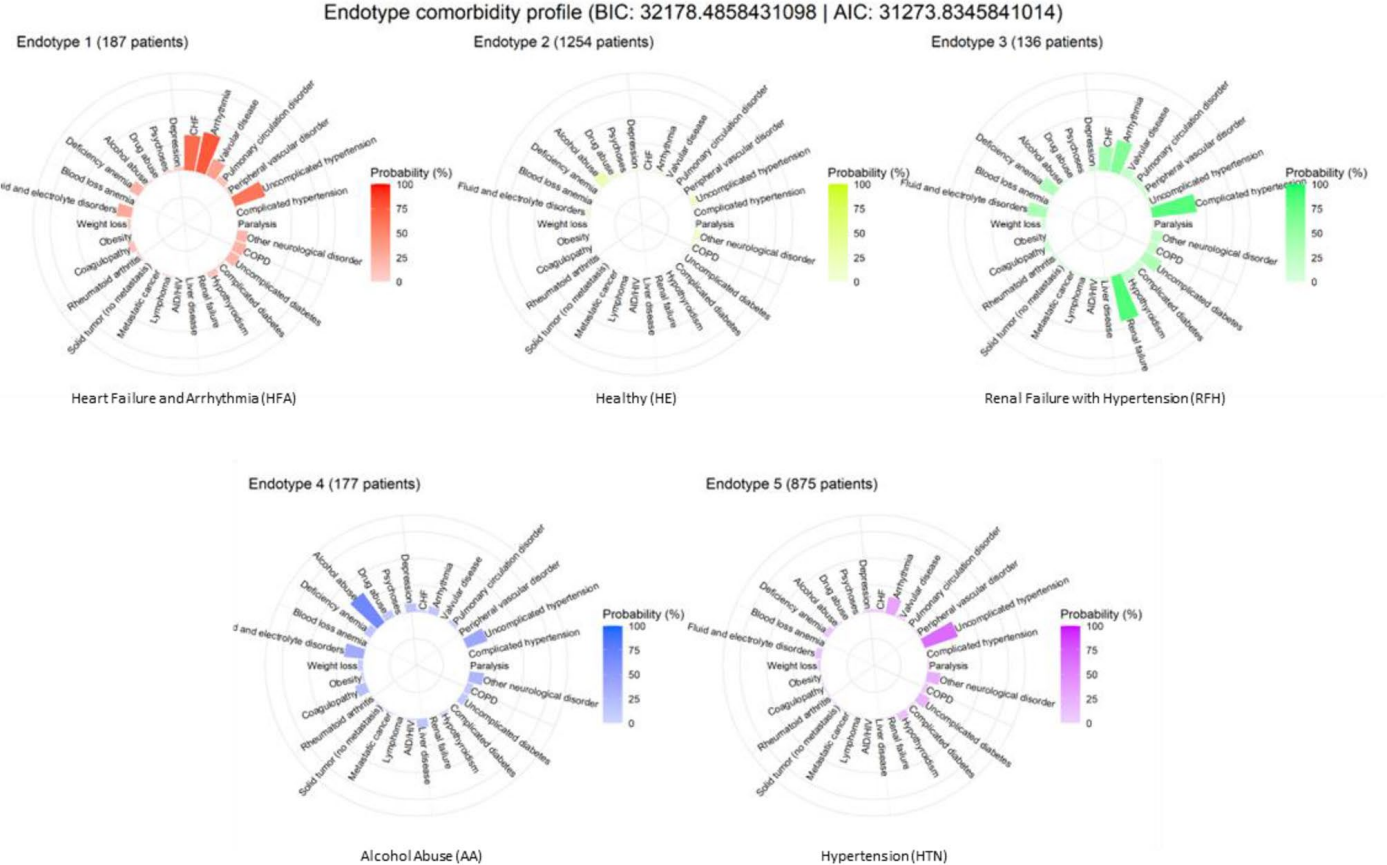
Comorbidity distribution of endotypes. Radial plots representing probabilities for each Elixhauser comorbidity within a particular endotype. A higher probability for an Elixhauser comorbidity for a given endotype means that a patient with this comorbidity has a higher chance of being classified in the given endotype. For example, a patient with only uncomplicated hypertension will be classified into Endotype 5 as it has the highest probability score for uncomplicated hypertension, higher than Endotype 1. However, if the patient also has congestive heart failure or arrhythmia, its score for Endotype 1 would be higher than for Endotype 5, hence resulting in them being categorized as such. Overall, these comorbidity distributions can be thought of as the probability of the prototypical patient in an endotype to have a specific comorbidity. Although similar, these values are not the same as the prevalence rates for comorbidities in an endotype. From these endotype plots, we can see common clinically distinct patient subtypes based on their presenting comorbidities. Based on these distributions, Endotype 1 was labeled Heart Failure and Arrhythmia (HFA), Endotype 2 labelled Healthy (HE), Endotype 3 labelled Renal Failure with Hypertension (RFH), Endotype 4 was labelled Alcohol Abuse (AA) and Endotype 5 labelled Hypertension (HTN).

We next analyzed the comorbidity data for the presence of endotypes using LCA. After 30 iterations, five stable clusters of patient endotypes emerged, detailed in Fig. 3 by their comorbidity probability distribution. Endotype 1 was labeled Heart Failure and Arrhythmia (HFA), characterized by cardiac comorbidities. Endotype 2 featured few comorbidities and was labelled Healthy (HE) and used as the reference endotype for subsequent analyses. Endotype 3 is characterized mainly by high rates of renal failure with complicated hypertension and was labelled Renal Failure with Hypertension (RFH). Endotype 4 was labelled Alcohol Abuse (AA), featuring high rates of alcohol abuse and relatively low rates of other comorbidities. Endotype 5 had high rates of hypertension and relatively low rates of other comorbidities, labelled Hypertension (HTN).

The distribution of TBI injury severity categories was relatively similar although HE and AA endotypes contained slightly larger proportions of severe TBI at 46.9% and 39.4%, respectively (Table 2). The HFA and RFH endotypes had nearly identical age distributions of mostly older individuals in addition to a small subpopulation of middle-aged individuals (17.9% middle-aged, 82.4% old vs 19.7% middle-aged, 80.9% old, respectively). The HTN endotype was composed mainly of older adults (68.6%) with a moderate proportion of middle-aged individuals (28.3%) and a small subpopulation of young individuals (3.7%). The AA group was primarily composed of middle-aged individuals (73.7%). HE endotype was composed of mainly young (47.6%) and middle-aged (40.0%) individuals. There were minor sex differences among a few endotypes, contributed to mainly by the young age segment which consistently had a higher proportion of male patients; middle-aged and old age groups across endotypes had comparable sex distributions (Supplementary Table 2). For comorbidity count, HE had the least number of comorbidities (0.8 ± 0.8) followed by HTN (2.5 ± 1.2) while the other comorbidities had higher averages within a similar range (AA: 4.0 ± 1.2, HFA: 4.5 ± 1.6, RFH: 5.5 ± 2.1).

**Table 2:** Demographics of endotypes.

Grouping	HFA	HE	RFH	AA	HTN
Mild	92 (50.0%)	454 (36.9%)	71 (53.8%)	71 (40.6%)	443 (51.6%)
Moderate	36 (19.6%)	200 (16.2%)	23 (17.4%)	35 (20.0%)	147 (17.1%)
Severe	56 (30.4%)	578 (46.9%)	38 (28.8%)	69 (39.4%)	269 (31.3%)
Young		586 (47.6%)		25 (14.3%)	32 (3.7%)
Middle aged	33 (17.9%)	493 (40.0%)	26 (19.7%)	129 (73.7%)	243 (28.3%)
Old	154 (82.4%)	172 (13.7%)	110 (80.9%)	23 (13.0%)	600 (68.6%)
Age (years)	80 ± 10	44 ± 20	79 ± 11	55 ± 15	74 ± 15
Number of comorbidities	4.5 ± 1.6	0.8 ± 0.8	5.5 ± 2.1	4.0 ± 1.2	2.5 ± 1.2

Average age, number of comorbidities, and breakdown of injury severity by Glasgow coma scale (GCS) and age categories across the five stable comorbidity endotypes. HFA: Heart failure and arrhythmia, HE: Healthy, RFH: Renal failure with hypertension, AA: Alcohol abuse, HTN: Hypertension. Young: 16–39, Middle-aged: 40–69, Old: 70 + . Mild: GCS 14–15, Moderate: GCS 9–13, Severe: GCS 3–8.

Each endotype had distinct rates of survival to discharge (Table 3). HE had the highest survival to discharge, followed by AA, HTN, RFH, and HFA. Survival differences between all endotypes were statistically significant (HE 78.5%, AA 70.1%, HTN 51.4%, RFH 40.4%, HFA 27.8%, *p* < 0.05 for all comparisons). RFH had the lowest rate of neurosurgical intervention (13.2%) which was statistically lower than HFA (27.3%) and HTN (25.7%) which had the highest intervention rates (RFH vs HFA *p* = 0.033, RFH vs HTN *p* = 0.022). All other comparisons of intervention rates were non-significant between endotypes). For LOS, endotypes were split into long (HFA: 10.2 ± 7.2 days, RFH: 8.7 ± 6.0 days, AA: 10.6 ± 7.5 days,) and short (HE: 7.3 ± 6.9 days, HTN: 7.7 ± 6.4 days) LOS groups in where *p* < 0.05 for comparisons between endotypes across groups and *p* > 0.05 for comparisons of endotypes within a group.

**Table 3:** Outcomes of endotypes.

Outcome	HFA	HE	RFH	AA	HTN
Alive	52	984	55	124	450
Expired	135	270	81	53	425
Survival rate (%)	27.8	78.5	40.4	70.1	51.4
Neurosurgical Intervention	51	269	18	41	225
No Neurosurgical Intervention	136	985	118	136	650
Intervention rate (%)	27.3	21.5	13.2	23.2	25.7
LOS (Days)	10.2 ± 7.2	7.3 ± 6.9	8.7 ± 6.0	10.6 ± 7.5	7.7 ± 6.4

Outcomes of survival rate, neurosurgical intervention rate, and length of hospital stay (LOS) across the five stable comorbidity endotypes without any stratification. HFA: Heart failure and arrhythmia

*HE* Healthy, *RFH* Renal failure with hypertension, *AA* Alcohol abuse, *HTN* Hypertension.

### Survival to discharge

Multivariable logistic regression models accounting for age, sex, and GCS showed that the endotypes HE, AA, and HTN had the highest survival rates (Table 4). Using the HE endotype as reference, AA (OR = 0.999, CI: 0.935–1.066) and HTN (OR = 0.962, CI: 0.919–1.007) had statistically similar survival rates compared to HE. HFA had the lowest survival rate (OR with respect to HE = 0.803, CI: 0.747–0.862) which was statistically different from all other endotypes. RFH had lower survival than HE (OR with respect to HE = 0.889, CI: 0.821–0.964) but higher than HFA (OR with respect to HFA = 1.108, CI: 1.011–1.214). Survival rates when accounting for age, sex, and GCS are as such: HE, AA, HTN > RFH > HFA. In contrast to the initial analyses, comparison of survival rates between HE, AA, and HTN becomes statistically insignificant when including age (OR per increase in age = 0.991, CI: 0.991–0.992) and GCS (OR per increase in GCS score = 1.028, CI: 1.024–1.031).

**Table 4:** Adjusted comparison of endotype survival outcomes.

Model	Variable	Odds Ratio	95% CI	*P*-value
HE as reference	HFA	0.803	0.747–0.862	< 0.001***
	RFH	0.889	0.821–0.964	0.004**
	AA	0.999	0.935–1.066	0.968
	HTN	0.962	0.919–1.007	0.095
	Age (years)	0.991	0.991–0.992	< 0.001***
	GCS score	1.028	1.024–1.031	< 0.001***
	Male sex	0.999	0.966–1.033	0.944
HFA as reference	HE	1.246	1.159–1.339	< 0.001***
	RFH	1.108	1.011–1.214	0.0284*
	AA	1.244	1.139–1.359	< 0.001***
	HTN	1.198	1.123–1.279	< 0.001***
RFH as reference	HFA	0.903	0.824–0.989	0.028*
	HE	1.125	1.038–1.219	0.004**
	AA	1.123	1.021–1.235	0.017*
	HTN	1.082	1.004–1.166	0.040*
AA as reference	HFA	0.804	0.736–0.878	< 0.001***
	HE	1.001	0.938–1.069	0.968
	RFH	0.890	0.810–0.979	0.017*
	HTN	0.963	0.899–1.032	0.288
HTN as reference	HFA	0.834	0.782–0.891	< 0.001***
	HE	1.040	0.993–1.088	0.095
	RFH	0.924	0.857–0.996	0.040*
	AA	1.038	0.969–1.112	0.288

Comparison of survival rates across the five stable comorbidity endotypes after adjustment by age, sex, and Glasgow coma scale (GCS) using multivariate logistic regression. The base regression model uses the HE endotype as the reference for the endotype variable in survival odds ratio calculations. Models using the other endotypes as reference are also reported for comparison. Odds ratio for age, sex, and GCS are omitted in these models as they remained constant for all models.

*HFA*: Heart failure and arrhythmia, *HE* Healthy, *RFH* Renal failure with hypertension, *AA* Alcohol abuse, *HTN* Hypertension.

Significance codes: “***”: ≤ 0.001, “**”: ≤ 0.01, “*”: ≤ 0.05.

Additional investigation of these three endotypes based on stratification by age and GCS revealed that HE had higher survival in the middle-aged group compared to AA (80.3% vs 69.8%, *p* = 0.010) and was similar in all age groups in comparison to HTN (Table 5). However, when further sub-stratifying age groups by GCS, some substrata comparisons become significant: HE has a higher survival rate than AA for the mild GCS group in both young (97.5% vs 83.3%, *p* = 0.043) and middle-aged groups (94.1% vs 70.0%, *p* < 0.001). Although survival was not different across age strata between HE and HTN, sub-stratification by GCS revealed that HE had better survival for the mild (94.1% vs 87.2%, *p* = 0.043) and severe (66.7% vs 52.6%, p = 0.043) GCS groups within the middle-aged group.

**Table 5:** Stratified endotype survival outcomes.

Age	TBI severity	Outcome	HE	AA	HTN
Young	Mild	Alive	193	5	6
		Expired	5	1	1
		Survival rate (%)	97.5	83.3	85.7
	Moderate	Alive	84	4	2
		Expired	5	1	0
		Survival rate (%)	94.4	80.0	100.0
	Severe	Alive	237	11	18
		Expired	56	3	4
		Survival rate (%)	80.9	78.6	81.8
Middle-aged	Mild	Alive	177	35	109
		Expired	11	15	16
		Survival rate (%)	94.1	70.0	87.2
	Moderate	Alive	73	21	28
		Expired	12	7	7
		Survival rate (%)	85.9	75.0	80.0
	Severe	Alive	140	33	40
		Expired	70	16	36
		Survival rate (%)	66.7	67.3	52.6
Old	Mild	Alive	38	10	158
		Expired	30	5	153
		Survival rate (%)	55.9	66.7	50.8
	Moderate	Alive	11	1	39
		Expired	15	1	71
		Survival rate (%)	42.3	50.0	35.5
	Severe	Alive	19	3	41
		Expired	56	3	130
		Survival rate (%)	25.3	50.0	24.0

Comparison of survival after stratification by both injury severity and for Healthy (HE), Alcohol abuse (AA), and Hypertension (HTN) endotypes. Only HE, AA, and HTN were chosen for additional stratified analysis as results from regression with adjustment by age and Glasgow coma scale (GCS) differed from initial results and due to significant variations of GCS and age distributions between these three endotypes. Young: 16–39, Middle-aged: 40–69, Old: 70 + . Mild: GCS 14–15, Moderate: GCS 9–13, Severe: GCS 3–8.

### Neurosurgical intervention

In comparison to HE, HFA (OR = 1.146, CI: 1.067–1.231) and HTN (OR = 1.123, CI: 1.074–1.175) had the highest rate of neurosurgical intervention while RFH (OR = 1.005, CI: 0.928–1.089) and AA (OR = 1.037, CI: 0.972–1.107) had statistically similar rates of intervention after adjusting for age, sex, and GCS (Table 6). Overall, results from the multivariable logistic regression divide the endotypes into the low (HE, RFH, AA) and high (HFA, HTN) intervention groups where rates are statistically different compared to endotypes of the other group for comparisons) while being statistically similar to endotypes of the same group.

**Table 6:** Adjusted comparison of endotype intervention rates.

Model	Variable	Odds Ratio	95% CI	*P*-value
HE as reference	HFA	1.146	1.067–1.231	< 0.001***
	RFH	1.005	0.928–1.089	0.893
	AA	1.037	0.972–1.107	0.272
	HTN	1.123	1.074–1.175	< 0.001***
	Age (years)	0.999	0.998–1.000	0.034*
	GCS score	0.978	0.975–0.982	< 0.001***
	Male sex	1.063	1.029–1.099	< 0.001***
HFA as reference	HE	0.872	0.812–0.937	< 0.001***
	RFH	0.877	0.801–0.961	0.005**
	AA	0.905	0.829–0.987	0.025*
	HTN	0.980	0.918–1.046	0.543
RFH as reference	HFA	1.140	1.041–1.248	0.005**
	HE	0.995	0.918–1.077	0.893
	AA	1.032	0.939–1.134	0.519
	HTN	1.117	1.037–1.204	0.004**
AA as reference	HFA	1.105	1.013–1.206	0.025*
	HE	0.964	0.903–1.029	0.272
	RFH	0.969	0.882–1.065	0.519
	HTN	1.083	1.011–1.16	0.022*
HTN as reference	HFA	1.020	0.956–1.089	0.543
	HE	0.890	0.851–0.931	< 0.001***
	RFH	0.895	0.831–0.964	0.004**
	AA	0.923	0.862–0.989	0.022*

Comparison of rates of neurosurgical intervention across the five stable comorbidity endotypes after adjustment by age, sex, and Glasgow coma scale (GCS) using multivariate logistic regression. The base regression model uses the Healthy (HE) endotype as the reference for the endotype variable in intervention rate odds ratio calculations. Models using the other endotypes as reference are also reported for comparison. Odds ratio for age, sex, and GCS are omitted in these models as they remained constant for all models.

*HFA* Heart failure and arrhythmia, *RFH* Renal failure with hypertension, *AA* Alcohol abuse, *HTN* Hypertension.

Significance codes: “***”: ≤ 0.001, “**”: ≤ 0.01, “*”: ≤ 0.05.

### Length of stay

Using ANCOVA to adjust for the covariates of age, sex, and GCS, HE had shorter LOS than HFA (difference = 4.6 ± 0.9 days, *p* < 0.001), RFH (difference = 3.3 ± 1.1 days, *p* = 0.015), and AA (different = 4.8 ± 0.9 days, *p* < 0.001) but had similar LOS compared to HTN (difference = 1.2 ± 0.6 days, *p* = 0.214) (Table 7). HFA and AA had almost identical LOS (difference = 0.2 ± 1.2 days, *p* = 1.000). LOS for the RFH endotype was statistically comparable to every other endotype (*p* > 0.05 for all comparisons) except HE. Overall, HE and HTN had the shortest LOS while HFA and AA had the longest LOS of the endotypes after accounting for age, sex, and GCS.

**Table 7:** Adjusted comparisons for endotype length of stay.

Variable	Df	Sum of squares	Mean square	F-value	*P*-value
Endotype	4	4430	1108	9.896	< 0.001***
Age (years)	1	1881	1881	16.807	< 0.001***
GCS	1	13,656	13,656	122.012	< 0.001***
Sex	1	593	593	5.296	0.022*
Residuals	2574	288,080	112		

Comparison hypothesis	LOS difference (days)	Standard error (days)	t-value	*P*-value	
HFA − HE = 0	4.6	0.9	4.815	< 0.001***	
RFH − HE = 0	3.3	1.1	3.101	0.015*	
AA − HE = 0	4.8	0.9	5.556	< 0.001***	
HTN − HE = 0	1.2	0.6	2.083	0.214	
RFH − HFA = 0	− 1.3	1.2	− 1.059	0.816	
AA − HFA = 0	0.2	1.2	0.210	1.000	
HTN − HFA = 0	− 3.3	0.9	− 3.845	0.001**	
AA − RFH = 0	1.5	1.3	1.214	0.729	
HTN − RFH = 0	− 2.0	1.0	− 2.055	0.226	
HTN − AA = 0	− 3.6	0.9	− 3.912	< 0.001***	

Comparison length of stay (LOS) across the five stable comorbidity endotypes after adjustment by age, sex, and Glasgow coma scale (GCS) using analysis of covariance (ANCOVA). ANCOVA model output and results of Tukey post-hoc comparisons for LOS between each pair combination of endotypes are reported below

*HFA* Heart failure and arrhythmia, *HE* Healthy, *RFH* Renal failure with hypertension, *AA* Alcohol abuse, *HTN* Hypertension.

Significance codes: “***”: ≤ 0.001, “**”: ≤ 0.01, “*”: ≤ 0.05.

### Predictive value of comorbidities and endotypes for survival

Logistic regression using all 30 Elixhauser comorbidity categories to predict survival to discharge accounts for ~ 15.3% of the variation in outcome (F(30, 2598) = 15.59, R^2^ = 0.153, *p* < 0.001) and had an AUC of 0.73. A simpler logistic regression using the five derived comorbidity endotypes accounted for 11.9% of the variation in survival (F(4, 2624) = 88.61, R^2^ = 0.119, *p* < 0.001) with an AUC of 0.69. Combining the base 30 Elixhauser categories with the endotypes in a full logistic regression model explains 17.0% of the variation in survival (F(34, 2594) = 15.57, R^2^ = 0.170, *p* < 0.001) and has an AUC of 0.74. The decrease in model error for predicting survival to discharge seen between the base logistic regression (including only the comorbidities) and the full model (including both comorbidities and endotypes) is statistically significant (F(4, 2594) = 13.23, *p* < 0.001).

With the inclusion of age, GCS, and sex into the logistic model using the 30 Elixhauser comorbidities, the model explains ~ 30.5% of the variation in survival (F(33, 2548) = 35.28, R^2^ = 0.305, *p* < 0.001) and had an AUC of 0.83. The addition of the five derived endotypes into this model results in explanation of ~ 31.7% of the variation in survival (F(37, 2544) = 31.91, R^2^ = 0.317, *p* < 0.001) with an AUC of 0.84. The decrease in model error in predicting survival after adding endotypes is statistically significant (F(4, 2544) = 3.13, *p* = 0.014). A model with age, GCS, sex, and comorbidity endotypes explains ~ 28.7% of survival variation (F(7, 2574) = 142.9, R^2^ = 0.287, *p* < 0.001) with an AUC of 0.82.For reference, a logistic model with only age, GCS, and sex accounts for 26.8% of the variation in survival with an AUC of 0.80. A model with only age accounts for 19.7% of survival variation with an AUC of 0.77; a model with only GCS accounts for 2.7% of survival variation with an AUC of 0.61; a model with only sex accounts for 0.9% of survival variation with an AUC of 0.55.

## Discussion

### Endotypes in TBI and other conditions

Our initial analyses are consistent with previous literature and clinical experience—that comorbidities are associated with clinical outcomes and that comorbidities tend to occur in patterns^44,45^.

In characterizing the relationship between comorbidities and clinical outcomes, we have identified five patient endotypes using LCA, based solely on the presence of medical conditions. These represent the most observed clinical patterns within the TBI population of the MIMIC III dataset. The notion of patient endotypes is a validated approach to precision medicine and has been used to elucidate heterogeneity in many other conditions such as sepsis and asthma^14,16,46^. In TBI, Åkerlund et al. suggested six endotypes in TBI patients based on GCS, body temperature, and lab values that added predictive value for in-hospital mortality^47^. This is the first study to consider endotypes in TBI patients utilizing comorbidities.

Our previous work^11^ investigated comorbidity endotypes within all of MIMIC III, identifying six overall endotypes while this current study focuses on the TBI population and produces similar but distinct endotypes. Both studies’ endotypes included hypertension, cardiac comorbidities, renal failure, and the lack of comorbidities (i.e., healthy). However, there were also major differences. While the AA endotype contained only alcohol use disorder as a comorbidity, the corresponding endotype in our previous analysis was “Hepatic addiction”, which, in addition to alcohol abuse, had significant rates of liver failure and coagulopathy. As well, the current analysis did not find any endotype that corresponded to the “Cardiopulmonary” endotype in our previous study, characterized by high rates of chronic pulmonary and cardiac comorbidities. Although similarities exist, these results suggest that distinct TBI comorbidity endotypes exist that differ from those of the general ICU population.

### Viewing TBI clinical outcomes by endotypes

Analyzing comorbidities via endotypes rather than individually shifts emphasis onto the fact that individual comorbidities don't exist in isolation and allows for a more comprehensive understanding of how a group of conditions affects the outcomes of acute injuries such as TBI. Additionally, it allows us to group patient presentations into clusters based on common pathophysiological patterns and clinical considerations which may facilitate decision-making.

#### Outcomes differences between medically complex endotypes

Heart failure comorbid with arrhythmia (HFA) and renal failure comorbid with hypertension (RFH) appear to be two common patterns of comorbidity in medically complex TBI patients. Overall, HFA has higher rates of neurosurgical intervention and mortality but similar LOS.

The association between renal failure and cardiac comorbidities is supported by many studies^48–51^ and may explain notable rates of congestive heart failure (CHF) and arrhythmia in the RFH comorbidity distribution. However, it is interesting that despite the notable presence of cardiac comorbidities in RFH, the pure cardiac endotype HFA, which has fewer comorbidities overall, still exhibited poorer outcomes. A large multicenter study by Shibahashi et al.^52^ found that both CHF and chronic kidney disease (CKD) increased the odds of in-hospital mortality for TBI patients. The odds ratio estimate for CKD (2.76, CI: 1.96–3.89) was notably higher than CHF (1.82, CI: 1.31–2.51), contrasting our results. However, Shibahashi et al. compared individual comorbidities while our analysis compares endotypes that contain multiple comorbidities at different rates. A possible explanation for the difference in our findings could be the notable prevalence of arrhythmias in the HFA and RFH endotypes. Patients with arrhythmias are frequently administered anticoagulant and/or antiplatelet medications, which may increase the need for intervention, risk of intervention, and mortality rates^53,54^. Hence, HFA having a higher rate of intervention and mortality may be contributed to by its higher rate of arrhythmia compared to the RFH endotype. Overall, our results suggest that the TBI patients of the cardiac-centric HFA endotype carry a poorer prognosis than the renal-centric RFH endotype. Both endotypes represent cohorts of older, clinically complex patients that fare significantly worse than the other endotypes even after controlling for age or injury severity. Future trials of TBI interventions may benefit from treating patients that fit into these endotypes as their own subgroups.

#### Alcohol abuse and length of stay

As alcohol abuse and hypertension are common in TBI patients^22^, we were interested in how the AA and HTN endotypes differed from HE. There is a robust relationship between alcohol use and TBI^55^ that is consistent with our finding that AA is a major TBI endotype. Overall, HE and AA had similar rates of survival and neurosurgical intervention but notably higher LOS. There have been many factors linking alcohol use disorder and increased LOS such as alcohol withdrawal including seizure^56–58^, increased risk of hospital complications and infection, particularly of pneumonia^59^, and pre-injury cerebral atrophy secondary to neurotoxicity^60,61^. Taken together, a slow and potentially turbulent recovery may explain the increased LOS in AA. Overall, we see that the AA endotype identified via LCA is consistent with previous literature and aligns with clinical observations.

#### Hypertension and outcomes of intervention and survival

We found that the HTN endotype has a higher rate of intervention compared to HE and a lower rate of survival, isolated to the middle-aged strata, particularly among mild and severe injury severities. Given that chronic hypertension is known to cause and accelerate cerebrovascular damage^62^, higher rates of adverse outcomes after TBI in the HTN endotype could be explained by a less compliant, and hence less resilient, remodelled cerebrovascular system^63,64^. The same mild injuries to HTN patients may result in greater damage and hence warrant a more aggressive treatment (e.g., ICP monitoring, craniotomy). The observation that differences in survival were only significant for the middle-aged group and not for the old age group may be due to the effect of age overshadowing the weaker effect of hypertension in this age group. While pre-hospital hypertension has been linked to increased TBI mortality^65,66^, a systematic review did not find chronic hypertension to increase in-hospital mortality in TBI patients^22^. These differences may result from other studies using the presence/absence of hypertension for their effect analysis in patients that potentially have other pre-existing comorbidities, contrasting our analysis that directly compares healthy patients with patients primarily with hypertension (without other comorbidities).

### Endotypes for precision medicine

LCA endotyping assesses the patient more holistically, accounting for both the presence and absence of diseases, to make comparisons between groups of patients that are similar. Linear regression attempts to describe the net effect of a single condition (e.g., hypertension) across a potentially heterogeneous population and struggles when input variables are correlated with each other, as is the case with comorbidities^67,68^. It also struggles to describe outcomes that cannot be explained by a linear combination of the model inputs whereas LCA endotypes have an aspect of non-linearity inherent to the complex relationships between input variables that they capture^69,70^. Our observed improvement in a logistic regression model's ability to predict survival by incorporating endotypes, in addition to individual comorbidities, supports the capturing of such additional non-linear effects through LCA. A large proportion of the endotypes’ predictive power for survival likely comes from their capture of patient age (e.g., patients with hypertension are likely to be older than those with alcohol abuse). Such is suggested by the large amount of variation in survival explained by age alone (19.7%) combined with the minor improvement in performance seen by adding endotypes as a variable to the base model of only age, GCS, and sex (R^2^: 26.8% to 28.0%; AUC: 0.80 to 0.82). Interestingly, our comorbidity endotypes have more predictive value as an isolated variable for survival than GCS (R^2^: 11.9%vs 2.7%, AUC: 0.69 vs 0.61) suggesting that patient-inherent factors may be more important to TBI outcomes than initial injury severity as measured by GCS.

As our endotypes involved only broad comorbidity variables, the overall improved predictability offered by endotypes in our analysis was relatively modest (~ 2% in model fit by R^2^, ~ 2% in model AUC). This improvement may be larger for complex endotypes that integrate continuous variables, especially those exhibiting a parabolic association with outcomes such as blood pressure, hemoglobin, and temperature. Therefore, endotype analysis offers a different perspective on the data that may be better suited to precision medicine and prognostication than regression with individual variables.

### Limitations

LCA is a method that attempts to deduce a possible explanation given the observed data points. A common concern with LCA is that results invite interpretation despite the lack of a true reasonable interpretation in reality^23,71^. Therefore, it relies on the researcher’s judgement to determine if the latent classes represent meaningful entities which introduce a degree of interpretive bias into the analysis. Through our cluster selection process, we have attempted to ensure that the final set of clusters represented the simplest clinical explanation, but we cannot exclude the existence of a potentially more complex set of endotypes that better explain the variance in TBI patient comorbidities.

While the LCA endotypes help explain the variation of comorbidities across patients, there also exists more layers of variation within each identified endotype, as not all patients within endotypes are identical. We try to account for this in the analysis of our primary outcome of survival with the use of stratification by GCS and age to capture important variations common to all TBI patients. However, it could be argued that GCS and age have differential effects on outcomes depending on endotype and that there may be additional condition-specific factors to consider for each endotype. For example, creatinine measures may be relevant in RFH to account for the extent of renal failure while total cumulative alcohol intake could be used to stratify AA patients into different exposure levels. Unfortunately, due to limited granularity, and potentially limited sample size in substrata, we did not investigate this further. This approach of granular analysis could be a strategy for future research toward increasingly personalized medicine using big data.

Another limitation of this study is the size of the MIMIC III dataset. Although it is a large and comprehensive dataset, there is limited granularity that may not capture the full complexity of patient health or outcomes. We do not have clinical justifications behind decisions, such as neurosurgical intervention, which requires the context of individual patient risk assessment. The data is limited to a single center, which may not be generalizable to the overall population and may have policies and personnel preferences that introduce artifacts in variable coding. In particular, there may be inaccuracies and staff biases in the ICD9 codes that we have used for the implementation of Elixhauser comorbidities. The same analysis using another database may yield a set of comorbidity endotypes that differ in significant ways. Finally, limitations inherent to retrospective designs apply as well. Therefore, the next steps for our research will involve the replication of our methods on different TBI databases, and ultimately prospective studies, to assess the generalizability of our findings.

## Conclusion

TBI, like many other conditions, is complex with varied clinical and biological presentations that contribute to their heterogeneity. In this study, we demonstrate the discovery of TBI comorbidity endotypes with distinct clinical outcomes. Our results suggest that viewing clinical variables, such as the presence or absence of a disease, in combinations is a promising way to address and better understand the heterogeneity observed in TBI and other complex conditions. This study serves to expand the applicability of the growing concept of clinical endotypes by validating the approach in a highly prevalent condition such as TBI. By utilizing approaches such as LCA that can extract meaningful combinations from large volumes of data, we can move towards precision medicine in an era that is becoming increasingly data-rich.

### Supplementary Information

Supplementary Information.

## Data Availability

All code used for analysis and visualization of our data is available on GitHub: https://github.com/SteveHQiu/TBIClustering. Data used in our analysis were extracted from the Medical Information Mart for Intensive Care III (MIMIC-III) dataset which is a freely available database: https://physionet.org/content/mimiciii/1.4/.
