# Supplementary material for: Identification of clinically relevant patient endotypes in traumatic brain injury using latent class analysis
Source: Sci Rep. 2024 Jan 14;14:1294. doi: 10.1038/s41598-024-51474-0 (PMC10788338; doi:10.1038/s41598-024-51474-0)
Supplement: Supplementary file 1 — Supplementary Information. [file 41598_2024_51474_MOESM1_ESM.docx]

Supplementary Table 1: TBI ICD-9 Codes to identify patient cohort

Groups of ICD-9 codes used to define our TBI cohort from the MIMIC-III dataset. Adapted from ^1–3^.

| **Diagnosis** | **ICD-9 Code** |
| --- | --- |
| Skull fracture | 800.0 – 801.9, 803.0 – 804.9 |
| Intracranial contusion | 800.1, 800.6, 801.1, 801.6, 803.1, 803.6, 804.1, 804.6, 851.0 – 851.1, 851.4 – 851.5, 851.8 – 851.9 |
| Intracranial hemorrhage | 800.2 – 800.3, 800.7 – 800.8, 801.2 – 801.3, 801.7 – 801.8, 803.2 – 803.3, 803.7 – 803.8, 804.2 – 804.3, 804.7 – 804.8, 852.0 – 853.1 |
| Concussion | 850.1 – 850.5, 850.9 |
| Unspecified head injury | 800.3 – 800.4, 800.8 – 800.9, 801.3 – 801.4, 801.8 – 801.9, 803.0 – 803.9, 804.3 – 804.4, 804.8 – 804.9, 850.9, 851.8 – 851.9, 853.0 – 854.1, 959.01 |

Supplementary Figure 1: Relative risk ratio

$$RR_{ij}=\frac{C_{ij}N}{P_{i}P_{j}}, \sigma_{ij}=\frac{1}{C_{ij}}+\frac{1}{P_{i}P_{j}}-\frac{1}{N}-\frac{1}{N^{2}}, t=\frac{1-\left| RR_{ij} \right|}{\sigma_{ij}}$$

Where $RR_{ij}$ is the relative risk between condition $i$ and $j$, $C_{ij}$ is the total number of patients affected by both conditions $i$ and $j$, $N$ is the total patient population, $P_{i}$ and $P_{j}$ are the prevalence of conditions $i$ and $j$ in the patient population, and $\sigma_{ij}$ is the estimate of the standard deviation for the distribution of $RR_{ij}$ values. We can then approximate a t-test statistic for the given $RR_{ij}$ testing the null hypothesis $RR_{ij}=1.0$ using the standardized distance of $RR_{ij}$ from 1.0 and obtain a p-value from this test. Adapted from Hidalgo et al^4^.

Supplementary Figure 2: Cluster performance


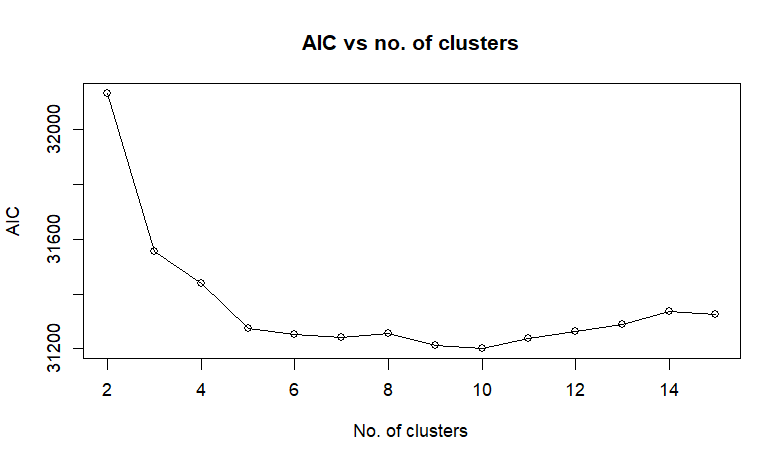


A graph representing the fit of the model (via Akaike information criterion (AIC) values) as a function of number of clusters included in the model. The inflection point of this graph helps to choose the best model that balances between accuracy (low AIC, better fit) and complexity (low number of clusters). Our algorithm calculates the inflection point to be at five clusters which represents the optimal number of clusters to have in a model.

Supplementary Figure 3: Comorbidity profiles of recurring endotypes in stability analysis


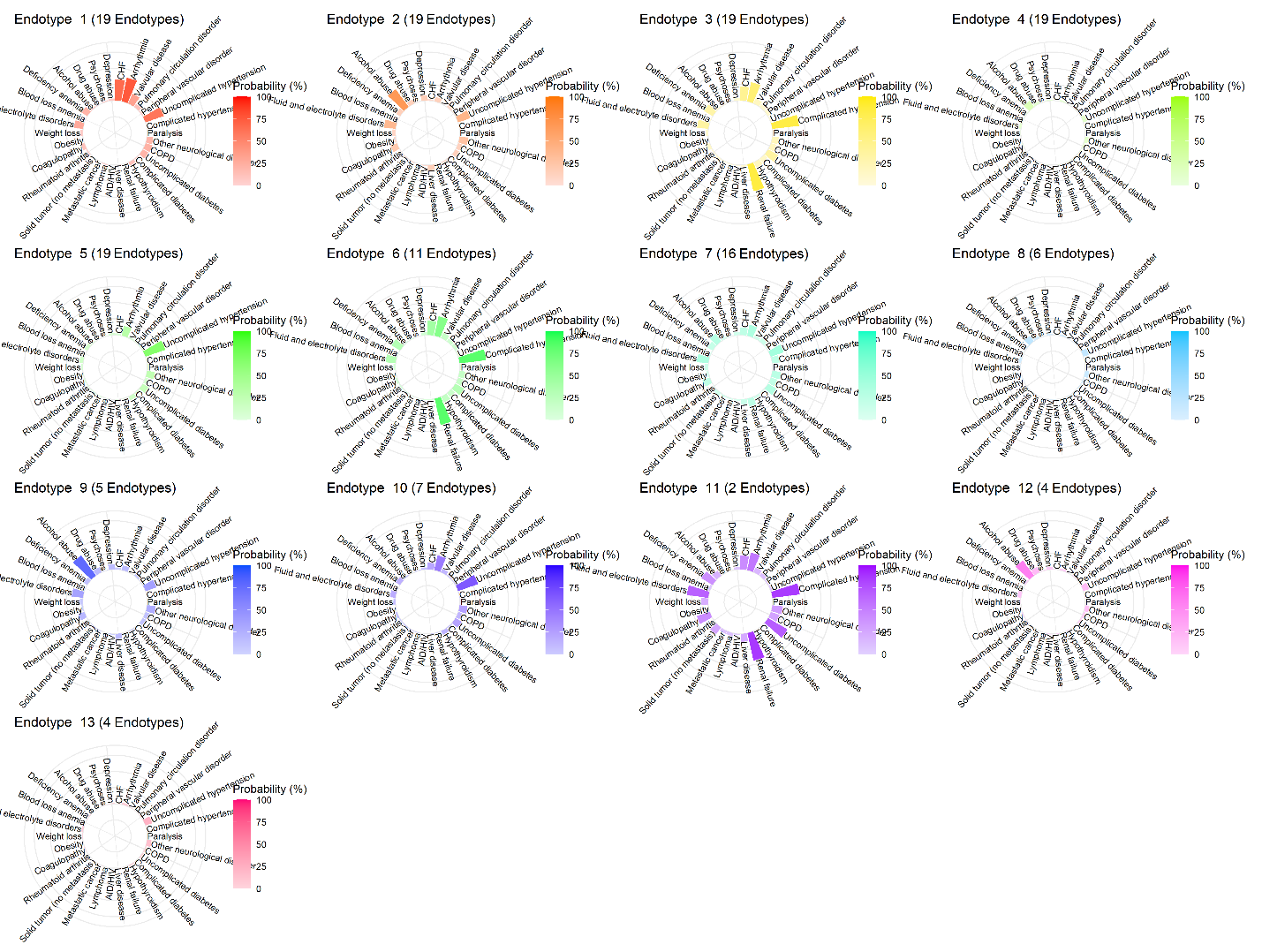


Comorbidity distribution of recurrent endotypes from 30 LCA models containing 5 clusters each. Notice that the top 5 most common recurrent endotypes all have the same number of clusters in them (19). This is due to 19 of the 30 LCA models having statistically identical clusters and hence considered part of the same recurrent endotype. Upon corroboration with each model's respective AIC values, we found that all 19 models had nearly identical low AIC values implying that these were the models with the "best fit". As such, the top 5 recurrent endotypes within this figure represent the most stable/accurate endotypes.

Supplementary Table 2: Sex distribution of each age segment across endotypes

Breakdown of the percentage of males in each age segment (young, middle-aged, and old) across the five stable endotypes. Except for within the young age segment where patients are primarily male, the sex proportion is mostly similar across endotypes for the middle-aged and old categories. HFA: Heart failure and arrythmia, HE: Healthy, RFH: Renal failure with hypertension, AA: Alcohol abuse, HTN: Hypertension. Young: 16-39, Middle-aged: 40-69, Old: 70+

|  | HFA | HE | RFH | AA | HTN |
| --- | --- | --- | --- | --- | --- |
| Young |  | 75.26% |  | 84.00% | 78.13% |
| Middle-aged | 63.64% | 69.57% | 50.00% | 68.99% | 61.73% |
| Old | 45.45% | 54.65% | 59.09% | 65.22% | 46.50% |

Supplementary Table 3: GCS across age groups and age across GCS groups of endotypes

Table characterizing the mean and standard deviation of GCS across age categories and age across GCS categories for each comorbidity endotype to ensure that GCS and age segments were comparable across endotypes. GCS scores across age groups were relatively uniform between endotypes. However, there were notable differences of age across GCS groups between endotypes, particularly that age was younger across HE and AA in most GCS groups compared to HFA, RFH, and HTN. HFA: Heart failure and arrythmia, HE: Healthy, RFH: Renal failure with hypertension, AA: Alcohol abuse, HTN: Hypertension. Young: 16-39, Middle-aged: 40-69, Old: 70+

| Characteristic | Strata | HFA | HE | RFH | AA | HTN |
| --- | --- | --- | --- | --- | --- | --- |
| GCS | Young |  | 9 ± 5 |  | 8 ± 5 | 8 ± 4 |
|  | Middle-aged | 12 ± 4 | 10 ± 5 | 11 ± 4 | 10 ± 4 | 11 ± 4 |
|  | Old | 11 ± 4 | 10 ± 5 | 12 ± 4 | 12 ± 4 | 11 ± 4 |
| Age | Mild | 80 ± 10 | 45 ± 20 | 80 ± 10 | 60 ± 14 | 75 ± 13 |
|  | Moderate | 79 ± 10 | 44 ± 20 | 77 ± 13 | 51 ± 13 | 76 ± 13 |
|  | Severe | 82 ± 11 | 42 ± 20 | 78 ± 12 | 51 ± 15 | 71 ± 17 |

# References

1. De Crescenzo, L. A., Gabella, B. A. & Johnson, J. Interrupted time series design to evaluate ICD-9-CM to ICD-10-CM coding changes on trends in Colorado emergency department visits related to traumatic brain injury. *Injury Epidemiology* **8**, 15 (2021).

2. Carroll, C. P., Cochran, J. A., Guse, C. E. & Wang, M. C. Are we underestimating the burden of traumatic brain injury? Surveillance of severe traumatic brain injury using centers for disease control International classification of disease, ninth revision, clinical modification, traumatic brain injury codes. *Neurosurgery* **71**, 1064–1070; discussion 1070 (2012).

3. WISH Injury-Related Traumatic Brain Injury ICD-9-CM Codes. *Wisconsin Department of Health Services* https://www.dhs.wisconsin.gov/wish/injury/tbi-codes.htm (2019).

4. Hidalgo, C. A., Blumm, N., Barabási, A.-L. & Christakis, N. A. A Dynamic Network Approach for the Study of Human Phenotypes. *PLOS Computational Biology* **5**, e1000353 (2009).
